# Tea marinating-induced improvement of quality in roasted chicken: The potential relationship between tea, flavor, and hazardous substances

**DOI:** 10.1016/j.fochx.2024.102033

**Published:** 2024-11-22

**Authors:** Ji Wang, Jing Che, Xu-Song Wang, Lei Qin, Xu-Hui Huang

**Affiliations:** SKL of Marine Food Processing & Safety Control, National Engineering Research Center of Seafood, Collaborative Innovation Center of Seafood Deep Processing, Dalian Technology Innovation Center for Chinese Prepared Food, School of Food Science and Technology, Dalian Polytechnic University, Dalian 116034, China

**Keywords:** Tea marinating, Chicken, High-temperature roasting, Flavor, Hazardous compounds

## Abstract

The levels of flavor compounds and hazardous compounds are important indicators for evaluating high-temperature roasted food. In this paper, the effect of tea pre-marination on non-volatile compounds, volatile compounds, and hazardous compounds in roasted chicken. The results showed that the total content of key umami non-volatile compounds in roasted chicken marinated with green tea, white tea, and black tea increased by 17.43 % to 100.11 %. The content of alkenes, alcohols, aldehydes, and ketones in high-temperature roasted chicken marinated with oolong tea and yellow tea was increased. Different teas had varying inhibitory effects on hazardous compounds in high-temperature roasted chicken, while green tea exhibited the highest inhibition efficacy. The inhibition rates of green tea for ACY, 5-HMF, HCAs, and PAHs were 72.12 %, 69.87 %, 92.49 %, and 14.92 %, respectively. Flavonoids in tea may play an important role in the flavor enhancement and hazardous compounds inhibition of high-temperature roasted chicken.

## Highlights

• The flavor compounds and hazardous compounds was monitored
• Marinating with tea can improve the flavor of roasted chicken
• Tea can inhibit hazards compounds in high-temperature roasted chicken
• The compounds contributing to the flavor and hazardous compounds were flavonoids

## 1 Introduction

Roasted meat, as a historical and popular cuisine, was favoured by consumers for its attractive flavor, texture and color. In China, there are many types of roasted chicken, among which the influential brands are Shandong Dezhou roasted chicken, Anhui Fuliji roasted chicken, Liaoning Goubangzi roasted chicken, Henan Daokou roasted chicken. Roasted chicken is made through a marinating, drying and roasting process (Zhang et al., 2022).

Flavor is an important attribute in determining the quality of roasted meat. Volatile and non-volatile flavor compounds can stimulate the senses of smell and taste to create a pleasurable gastronomic experience for consumers (Shen et al., 2023; Tian et al., 2020). The development of flavor in roasted meat is a complex process. Flavor development occurs mainly at the roasting stage. There are a variety of common roasting methods available, including iron oven-roasted, electric-roasted, and soil oven-roasted (Nie et al., 2024). Roasting not only preserves the original flavor of the chicken, but also promotes the creation of new and pleasing flavors. The main sources of flavor in roast meat are thermal processing-induced protein degradation, lipid oxidation, and the Maillard reaction (Liu et al., 2020). However, high-temperature roasting is associated with the creation of hazardous compounds, such as acrylamide (ACY), 5-hydroxymethylfurfural (5-HMF), heterocyclic amines (HCAs), and polycyclic aromatic hydrocarbons (PAHs), which have potential carcinogenic properties, which have potential carcinogenic properties (Kim et al., 2021; Li et al., 2024). Therefore, it is necessary to control the levels of these substances in barbecued meat to ensure consumer health. Marinating meat products in advance to enhance their flavor and reduce the formation of associated hazardous compounds has gained significant popularity in the realm of roasted meat preparation (Viegas et al., 2012). This method not only elevates the taste profile but also demonstrates a remarkable ability to mitigate the presence of hazardous compounds within these culinary delights. Daniels et al. (1995) found that the incorporation of spices into duck broth can enhance the release of umami amino acids in meat products. Gibis and Weiss (2010) found the inclusion of hibiscus extract in the marinade for fried beef patties resulted in a noteworthy decrease in the presence of MeIQx and a generally reduced level of total HCAs.

Tea is a popular beverage worldwide, known for its distinctive flavor that is enjoyed by a majority of consumers. According to the processing methods and degree of fermentation, tea can be classified into six categories: yellow, white, oolong, black, dark, and green. The processing methods of green tea, yellow tea, and oolong tea mainly involve fixation; black tea predominantly undergoes fermentation, exhibiting the highest level of fermentation among tea types; dark tea is predominantly semi-fermented; white tea, with the least processing steps, is crafted by withering and drying after the plucking of fresh leaves (Liang et al., 2021). Tea contains abundant polyphenolic compounds, such as catechins, flavonoids, and phenolic acids. These compounds have antioxidant properties that help fight free radicals and reduce the formation of hazardous compounds (Wang et al., 2018). The research examining how tea marinades impact the flavor and harmful substances in chicken roasted at high temperatures. In addition, identification of important compounds in tea marinades that could influence both flavor and harmful elements, is certainly worthy of our focus and investigation.

In this study, the taste-active compounds, hazardous compounds, and volatile compounds in high-temperature roasted chicken marinated with different teas were researched using UPLC-MS/MS and SPME-GC–MS. In addition, this study also analyzed the correlation between the key components in tea marinades and taste-active compounds, volatile compounds, and hazardous compounds in roasted chicken. The findings of this research can provide a way to strike a balance between reducing hazardous compounds and enhancing flavor.

## 2 Materials and methods

### 2.1 Materials and chemicals standards

Acetonitrile, methanol, n-hexane, methyl-tert-butyl-ether (MTBE), formic acid, and acetic acid, were all HPLC grade and acquired from Spectrum Chemical Manufacturing Corporation (Gardena, CA, USA) and Aladdin Reagent Co, Ltd. (Shanghai, China). Anhydrous magnesium sulfate and sodium chloride were acquired from Kermel Chemical Reagent Co. Ltd. (Tianjin, China). Folrisil SPE solid phase extraction column was obtained from Agile Technologies Co, Ltd. (Tianjin, China).

D_3_-DL-glutamic acid, Cytarabine-13C_3_–5′-monophosphate, D_3_-DL-alanine, and D_3_-DL-aspartic acid were procured from Aladdin Reagent Co, Ltd. (Shanghai, China). 4,8-Di-MeIQx-d3 were procured from TRC Chemical Ltd. (Toronto, Canada). D_9_-TMAO and acrylamide-d_3_ were procured from Sigma Aldrich (St. Louis, MO, USA). PAHs, including Naphthalene (Na), 3,4-Benzopyrene (BaP), Fluorene (F), Anthracene (Ant), Benzo[*ghi*]perylene (B[*ghi*]P), Chrysene (97 %, Chr), Benzo[*k*]fluoranthene solution (B[*k*]F), Fluoranthene (Flu), Benz[*a*]anthracene (BaA), Benzo[*b*]fluoranthene (98 %, B[*b*]F) were procured from Macklin Co, Ltd. (Shanghai, China).

Black tea, oolong tea, yellow tea, green tea, white tea, dark tea were bought at the flagship store of Eight Horses (Shenzhen, China). Chicken samples were purchased from Zheng Da Foods (Beijing, China).

### 2.2 Sample preparation

Five g of each type of tea was weighed separately and 250 mL of water were mixed and boiled. Keeping it boiling for 10 min, then filter out the tea leaves and let the marinades rest until it reaches room temperature. The chicken was marinated with tea marinade at a ratio of 1:1 (g/mL) at 4 °C for 4 h. Control samples were prepared by marinating the meat in water for 4 h at 4 °C. After marinating, the surface of the chicken was drain. Then, the chicken were roasted at 240 °C for 20 min using the oven. The roasted chicken was cooled to room temperature and stored at −20 °C for further study.

### 2.3 Sensory evaluation

Ethical permission for sensory research was not required in our institution (Dalian Polytechnic University). All participants signed an informed consent before the sensory evaluation. The rights and privacy of all participants were protected during the execution of the study. The selection criteria were usability assessment, interest in participating in the study, no aversion to chicken and tea, allergy or intolerance, and normal perception. Descriptive sensory analysis was conducted by 20 members (11 women and 9 men, ages ranging from 20 to 40 years) to assess the effect of tea curing on roasted meat products. The analysis encompasses the assessment of color, taste, aroma, and texture. They received systematic training and had a wealth of experience in the evaluation of foods. Sensory evaluation for each attribute was performed using a non-structured scale ranging from 1 to 5 points.

The sensory evaluation team underwent a training program divided into four sessions, each lasting one h, to ensure result repeatability. During the initial session, the panelists evaluated various aspects of the chicken sample, including its color, taste, aroma, and texture. They followed clear instructions to accurately describe their observations. During the second, redundant descriptive terms were eliminated and new properties for testing chicken samples were introduced. Third, all the selected attributes were evaluated on an unstructured scale of 1–5. Finally, the team members evaluated different attributes independently according to the representative samples provided, and they knew nothing about the samples before.

During the evaluation sessions, all samples were assigned a random numerical label. The team members were then presented with the samples and each member performed three evaluations.

### 2.4 Determination of *E*-tongues

Samples were prepared according to a previously reported procedure (Okumura et al., 2004). Ten g of samples were weighed and homogenized with 20 mL of ultrapure water for 10 min at 7000 rpm (Ultra Turrax homogenizer, IKA Co, Heidelberg, Germany). Following a 15 min immersion in an ultrasonic bath, 10 mL of n-hexane was introduced. Subsequently, the mixture was vortexed for 1 min and centrifuged at 10000 rpm for 10 min (4 °C). The resulting lower liquid layer was then carefully transferred to a new vial. Repeat the previous step. The lower liquid layer was adjusted to a volume of 100 mL with ultrapure water for subsequent analysis.

### 2.5 Determination of *E*-nose

Samples were prepared according to a previously reported procedure (Huang et al., 2019). The PEN 3 E-nose was utilized to measure the odor response value, employing ten distinct types of metal oxide semiconductors that correspond to various volatile compounds. The sensor-compound relationships were established as follows: W1S matched methane and related compounds, W2S matched alcohols, aldehydes, and ketones, W3S matched long-chain alkanes, W5S matched nitrogen compounds, W6S matched hydrocarbons, W1C matched aromatic benzene compounds, W3C matched aromatic compounds with ammonia, W5C matched short-chain alkanes, W1W matched inorganic sulfides, and W2W matched organic sulfur compounds and aromatic compounds. Before testing, the samples underwent preincubation. 5 g of sample were placed in 20 mL airtight vials. The testing conditions consisted of an incubation temperature of 50 ± 1 °C, and the measurement time was set at 100 s.

### 2.6 Determination of taste-active compounds

The samples were prepared following the methodology previously reported by our team (Xin et al., 2023). Taste-active compounds were analyzed using HPLC-MS/MS (LC-30AC, Shimadzu, Japan, 5500 Qtrap System, AB Sciex, America). The separation of target analytes was achieved on an Infinity Lab Poroshell 120 HILIC-Z column (2.1 mm × 150 mm, 2.7 μm particle size, Agilent). The mobile phase, elution gradient, and mass spectrometry conditions were set according to previous reports. Perform qualitative and quantitative analysis of taste-active compounds in the samples using the internal standard method.

### 2.7 Determination of volatile compounds

The samples were prepared following the methodology previously reported by our team Chen (Chen et al., 2023). The volatile compounds analysis was performed using the 5977 A-7890B GC–MS (Agilent Technologies, Santa Clara, CA, USA) system with an HP-5MS column (30 m × 0.25 mm × 0.25 μm, Agilent Technologies). The temperature gradient and mass spectrometry conditions were set according to previous reports.

N-alkanes standards (C7–C30) were determined to calculate linear retention indices of volatile compounds (Vandendool & Kratz, 1963). Identify the volatile compounds and match them with the standards in NIST 14 through mass fragmentation and RI. The relative quantification of volatile compounds was calculated using the internal standard (IS).

### 2.8 Determination of ACY, 5-HMF, and HCAs

The samples were prepared following the methodology previously reported by our team and some changes were made (Gao et al., 2021). Three g of samples were weighed and added internal standard (80 μL of 2 μg/mL acrylamide-d_3_ and 1 μg/mL 4,8-Di-MeIQx-d_3_). The samples were homogenized in 10 mL of ultrapure water and 10 mL of acetonitrile at 8500 r/min for 2 min. After 15 min of ultrasonication, 5 mL of n-hexane, 4 g of anhydrous magnesium sulfate, and 0.5 g of sodium chloride were subsequently added. After homogenization for 2 min, samples underwent centrifugation at 3040 *g* for 10 min. The acetonitrile layer was collected for 5 mL and subjected to evaporation using a high-speed vacuum concentrator. The resulting dried sample was reconstituted in water/acetonitrile (1:1, *v*/v) and agitated for 30 s. Following centrifugation at 20000 *g* (4 °C) for 10 min, the supernatant was extracted for subsequent analysis.

Quantification of HCAs, 5-HMF, and ACY were performed by 5500 Q TRAP. An Acquity UPLC HSS T3 column (2.1 mm × 100 mm, 1.8 μm), equipped with an Acquity UPLC HSS T3 Van Guard Pre-column (2.1 mm × 5 mm, 1.8 μm), was utilized to separate ACY, 5-HMF, and HCAs. The analytical column was maintained at 40 °*C. mobile* phase A was 0.1 % (*v*/v) formic acid in water; mobile phase B was 0.1 % (v/v) formic acid in acetonitrile. The following elution gradient was applied: 0–1.5 min, 1 % B; 1.5–16.5 min, 1–99 % B; 16.6–20 min, 99–1 % B. The flow rate was 0.3 mL/min. The injected volume was 1 μL. The solvent removal temperature was set to 600 °C. The entry potential and the ion spray voltage of the source were set at 10 and 5500 V. The curtain gas and Ion source were high-purity nitrogen at 35 and 60 psi. The content in the sample was analyzed by the ratio of peak height to the internal standard.

### 2.9 Determination of PAHs

Two g of sample were weighed and vortexed with 10 mL of acetonitrile and 10 mL of hexane saturated with acetonitrile. After ultrasonic for 15 min, the samples were homogenized at 8000 r/min (4 °C) for 10 min, and the acetonitrile was collected. 10 mL of acetonitrile was added and repeat the extraction step. The acetonitrile obtained from two extractions was evaporated to dryness by rotary evaporation. 5 mL of n-hexane was added to dissolve the residue. Five mL of n-hexane was transferred into a Florisil solid-phase extraction column that had been activated with 5 mL of dichloromethane and 10 mL of n-hexane. The chicken heart bottle was washed with 5 mL of n-hexane, and the washing solution was combined with the column. 8 mL mixture of n-hexane and dichloromethane (1:1) was used for elution. All eluates were collected and nearly dried by nitrogen blowing. The dried sample was dissolved in 1 mL of acetonitrile and shaken for 1 min by vertexing. Before analysis, the solution was filtered through a 0.22 mm membrane filter.

The PAHs were analyzed using Agilent 1260 Infinity-FLD (Agilent Technologies, Santa Clara, CA, USA). Waters PAH C18 column (4.6 mm × 250 mm, 5 μm) was performed to chromatographic analysis at 35 °C. The elution solvents consisted of mobile phase A (water) and mobile phase B (acetonitrile) at a flow rate of 1.2 mL/min. The following elution gradient was applied: 0–9 min, 60 % B; 9–12 min, 60–100 % B; 12–28 min, 100 % B; 28–32 min, 100–60 % B; 32–35 min, 60 % B. Sample injection volume was 10 μL. The excitation and emission wavelengths were 279 and 340 nm, 0.0–12.5 min; 248 and 375 nm, 12.5–13.65 min; 280 and 462 nm, 13.65–15.50 min; 270 and 385 nm, 15.50–17.50 min; 270 and 446 nm, 17.50–18.50 min; 292 and 410 nm, 18.50–25.70 min; 305 and 480 nm, 25.70–35.00 min. The sample's qualitative and quantitative assessment of PAHs was conducted through the utilization of an external calibration curve.

### 2.10 Determination of tea compositions

Ten g of tea samples was weighed separately and 500 mL of ultrapure water was weighed and boiled, the boiling state was maintained for 10 min. The solvent was evaporated nearly 100 mL and lyophilized. Seventy mg of samples was weighed, followed by the addition of 225 μL of chilled methanol, vortexed for 15 s. Then, 250 μL of ultrapure water was introduced and vortexed for 15 s. Finally, 750 μL of MTBE was added, and the mixture was vortexed for 6 min. After centrifuging at 8000 g for 10 min (4 °C), 200 μL of the lower liquid layer was collected and 600 μL of ice-cold methanol/isopropanol (1:1, *v*/v) was added. After mixing for 6 min, the mixture was centrifuged at 20,000 *g* for 2 min (4 °C), 300 μL of supernatant was collected and dried using a high-speed vacuum concentrator. 100 μL of methanol/water was used to redissolve the dried sample. The solution was shaken for 2 min and centrifuged 20,000 *g* for 2 min (4 °C) to collect impurities before analysis.

UPLC-Q Extractive X was used to evaluate the compounds of tea (Chen et al., 2023). To assess the detection accuracy and stability, QC samples and blank samples were set up for each of the 10 samples. The Acquity UPLC HSS T3 column (2.1 mm × 100 mm, 1.8 μm), equipped with an Acquity UPLC HSS T3 Van Guard Pre-column (2.1 mm × 5 mm, 1.8 μm), was used for chromatographic analysis at 25 °*C. mobile* phase A consisted of 0.1 % Formic acid in water, whereas mobile phase B consisted of 0.1 % Formic acid in acetonitrile at a flow of 0.5 mL/min. A linear gradient was used: 0–1 min, 1 % B; 1–8 min, 1–99 % B; 8–10 min, 99 % B; 10 min, 99–1 %B; 10–12 min, 1 % B. The injection volume was 1 μL. ESI was used to carry out electrospray ionization. The detection was performed in positive mode, and mass spectrometric data were acquired over the range of 120–1200 *m*/*z*. The sheath gas flow rate was 60 %, the aux gas flow rate was 25 %, the purge gas flow rate was 2 %. The spray voltage was at 3.6 kV, the capillary temperature was 380 °C, aux gas heater temperature was 370 °C. The metabolites were annotated by MS-DIAL software, HMDB (http://www.hmdb.ca/) based on accurate mass, and fragment matching. Peak intensities were used to show metabolite trends.

### 2.11 Statistical analysis

All experiments were replicated three times. The data were organized using Microsoft Office 2016, and statistical analyses were performed using the SPSS 20.0 software. ANOVA was used to statistically assess the outcomes (*p* < 0.05). Metabo Analyst 5.0 was used to carry out VIP score (variable importance in projection), heatmap, PLS-DA (partial least squares discriminant analysis), and PCA (principal component analysis) (Chong et al., 2018). Prismchs was used for boxplots. TBtools is used for correlation heatmap. The Xlstat was used for PLS-R (Partial least squares regression) analysis.

## 3 Results and discussion

### 3.1 Sensory evaluation, *E*-nose, and *E*-tongue of chicken marinated with different teas after high-temperature roasting

Variations in the color and flavor of roasted chicken were observed after marinated with tea, as analyzed in Table 1. Black tea and yellow tea resulted in lower “color” scores compared to the control sample, while oolong tea showed higher “color” scores. This difference may be due to the darker color of black tea and yellow tea, a slightly inferior color in the marinated roasted chicken. The “aroma” score of roasted chicken marinated with white tea was lower than the control sample, whereas black tea, oolong tea, and green tea enhanced the aroma. Oolong tea, yellow tea, and green tea improved the “taste” of high-temperature roasted chicken. All tea marinades improved the texture of the chicken, but the variations were not statistically significant, indicating no significant differences.

**Table 1:** Sensory evaluation score of roasted chicken at high temperature after marinating with distilled water and different tea marinades.

	Control	Black tea	Oolong tea	Yellow tea	Green tea	White tea	Dark tea
Color	3.70 ± 0.98^ab^	2.80 ± 0.95^c^	3.85 ± 0.93^a^	3.10 ± 0.79^bc^	3.80 ± 0.89^ab^	3.70 ± 0.92^ab^	3.30 ± 0.92^bc^
Aroma	3.30 ± 1.13^ab^	3.75 ± 1.02^a^	3.70 ± 0.66^a^	3.35 ± 0.67^ab^	3.70 ± 1.08^a^	2.95 ± 0.89^b^	3.10 ± 0.85^ab^
Taste	3.20 ± 1.20^a^	3.15 ± 0.88^a^	3.50 ± 0.95^a^	3.25 ± 1.02^a^	3.40 ± 1.27^a^	3.20 ± 1.06^a^	3.05 ± 1.00^a^
Texture	2.85 ± 1.14^a^	2.85 ± 1.18^a^	3.45 ± 0.89^a^	3.45 ± 0.89^a^	3.05 ± 0.94^a^	3.05 ± 0.94^a^	2.95 ± 0.89^a^

Values of different groups with lower-case letters (a-c) is significantly different at *P* < 0.05.

**Fig. 1:**
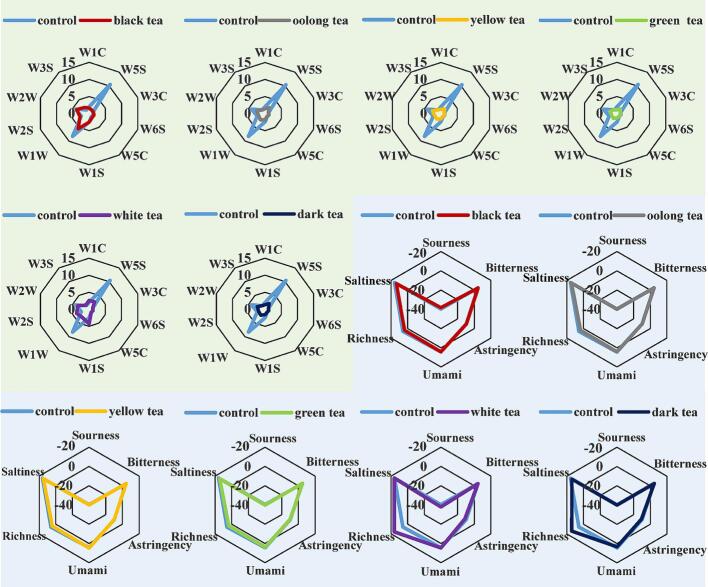
Radar chart of *E*-nose response and E-tongue response of chicken roasted at high temperature after marinated with tea. The compounds in the green area are radar charts of E-nose response. The compounds in the blue area are radar charts of E-tongue response. (For interpretation of the references to color in this figure legend, the reader is referred to the web version of this article.)

The *E*-nose response of chicken marinated with different teas after high-temperature roasting is shown in Fig. 1. The volatile components, including long-chain alkanes (W3S), nitrogen-containing aromatic compounds (W3C), hydrides (W6S), and short-chain alkanes (W5C), remained consistent in the marinated chicken after roasted, with minimal influence from the different tea marinades. While alkane compounds contribute a pleasant aroma to the samples, their impact on the overall flavor is limited due to their high odor threshold. The nitrogen compounds (W5S) and inorganic sulfides (W1W) demonstrated significant responses in the control samples, while the responses in other tea marinated chicken samples showed varying degrees of reduction. This result is similar to that reported by Sun, Yu, Wang, et al. (2024), who found higher response values for sensors W5S, W1S, W1W, W2S, W2W in chicken. Inorganic sulfides may undergo reactions with precursor compounds in food, leading to the formation of deleterious compounds. This could be attributed to the higher concentration of hazardous compounds in the control samples. Chicken samples marinated with oolong tea, yellow tea, green tea, and black tea exhibited similar characteristics.

The *E*-tongue response of chicken marinated with different teas after high-temperature roasting is shown in Fig. 1. The negative response values of the sensors indicate their minimal contribution to the overall taste. Sourness and astringency had a limited impact on the taste changes of chicken marinated with different teas after high-temperature roasting. Saltiness played the most significant role in overall taste perception. Marinated with black, oolong, yellow, green, and black tea effectively reduced the saltiness in high-temperature roasted chicken. The chicken marinated with yellow tea and white tea showed a higher response for umami compared to control samples. Marinated with white tea and black tea significantly enhances the richness of high-temperature roasted chicken. The flavor variances may be ascribed to the existence of unique flavor compounds in tea, consequently impacting the aroma of high-temperature roasted chicken marinated with tea. Tea polyphenols, as a predominant constituent in tea leaves, exhibit antioxidative properties. During the processes of marination and roasting, tea polyphenols engage in reactions with proteins, fats, and other constituents in the ingredients, giving rise to the formation of organic acids, alcohols, and other substances, consequently affecting the taste and aroma (Bortolini et al., 2021).

### 3.2 Taste-active compounds in chicken marinated with different teas after high-temperature roasting

**Fig. 2:**
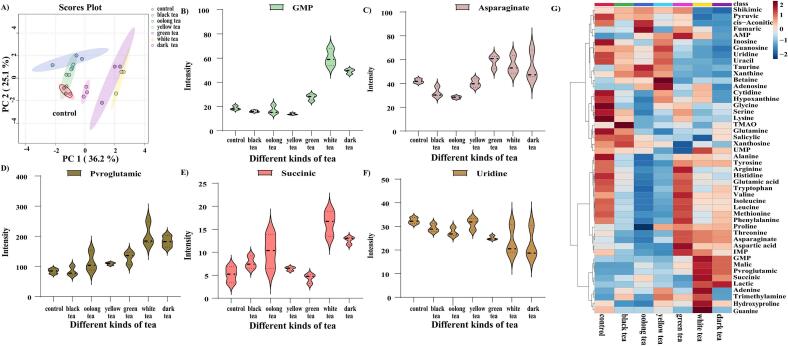
Changes of taste-active compounds in chicken roasted at high temperature after marinating with different teas. (A) The PCA of taste-active compounds. (B)-(F) Intensity changes of some important taste-active compounds. (G) The variable importance in the projection scores of taste-active compounds.

The results of the PCA (Fig. 2A) showed that the different types of tea marinades have varying degrees of influence on the taste-active compounds of high-temperature roasted chicken. The taste-active compounds of high-temperature roasted chicken marinated with water and black tea are similar, but the content is slightly different. Green tea exhibited distinct separation from the other samples, indicating significant differences in the taste-active compounds of high-temperature roasted chicken marinated with green tea compared to other tea marinades. The differences in taste-active compounds of roast chicken marinated with oolong tea, yellow tea and black tea were relatively small.

Fig. 2G represents the heatmap of taste-active compounds change in high-temperature roasted chicken marinated with different teas. High-temperature processed foods contain a significant amount of FAA, which serves as the principal flavor compound in these food products. FAA contributes to various taste sensations, including bitterness, sweetness, umami, and tastelessness. The tea marinade possesses antioxidant properties, effectively scavenging free radicals, and mitigating lipid and protein oxidation in high-temperature roasted chicken. It can inhibit the oxidative degradation of amino acids (Yao et al., 2021). In addition, tea contains large amounts of amino acids. The accumulation of amino acids makes an important contribution to flavor. It can be observed that the bitter-tasting amino acids such as methionine were significantly reduced in high-temperature roasted chicken marinated with teas. Aspartic acid, an umami-tasting amino acid, increases in high-temperature roasted chicken marinated with green tea, white tea, and black tea. Aspartic acid is involved in human tricarboxylic acid metabolism and is frequently utilized in medical interventions for conditions like hypertension. In high-temperature roasted chicken marinated with green tea, white tea, and black tea, the content of sweet-tasting amino acids such as serine and threonine increases.

Apart from FAA, nucleotides and related chemicals are significant non-volatile taste-active compounds. IMP, GMP, AMP, UMP, and CM are crucial umami flavor components in meat products (Yue et al., 2016). The AMP content in high-temperature roasted chicken experienced an increase after being marinated with tea, thereby enhancing the umami taste or interacting with other compounds. This phenomenon could be attributed to the impact of tea marination on the generation and degradation of nucleotides in high-temperature roasted chicken. AMP, a metabolite of ATP in normal biological metabolism, degrades to IMP, which is further metabolized to Ino and Hx through phosphatase metabolism (Feng et al., 2016). IMP is considered a major precursor in raw meat as it is one of the compounds derived from ATP degradation (Rotola-Pukkila et al., 2015). After being marinated with green tea or white tea marinade, the content of IMP increases in high-temperature roasted chicken. Compounds such as Inosine, Hypoxanthine, Adenine, Adenosine, Guanosine, and Xanthine are metabolites of nucleotides and typically possess a bitter taste. Tea marinating reduces the levels of Ino and Hx in high-temperature roasted chicken.

Ten different compounds (VIP > 1.0) that differential the taste of high-temperature roasted chicken marinated with various teas were identified. These compounds include cis-Aconitic, Malic, GMP, Pvroglutamic, Salicylic, Pyruvic, Taurine, Succinic, Asparaginate, and Uridine. These compounds primarily affect the umami and astringent tastes of high-temperature roasted chicken. GMP (Fig. 2B), as one of the major nucleotides, significantly affects cellular metabolism, contributes to meat flavor, and works in conjunction with IMP to stimulate umami production (Dai et al., 2011). Pvroglutamic (Fig. 2D), an important organic acid, imparts a distinctive umami and sour taste (Chen & Zhang, 2007). GMP, Asparaginate (Fig. 2C), and Succinic (Fig. 2E) were umami taste-active compounds that caused differences among the components, with their total amounts in high-temperature roasted chicken following the order: white > dark > green > water > yellow > black > oolong. Uridine (Fig. 2F) is a key compound for the astringent taste, and its level decreases in high-temperature roasted chicken after tea marinated.

### 3.3 Volatile compounds in chicken marinated with different teas after high-temperature roasting

**Fig. 3:**
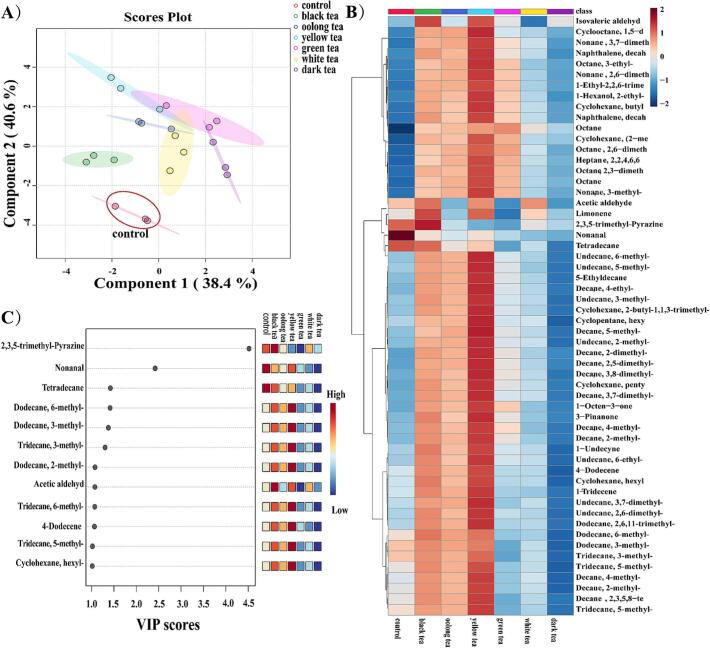
Changes of volatile compounds in chicken roasted at high temperature after marinating with different teas. (A) The PLS-DA of volatile compounds. (B) Heatmap of volatile compounds. (C) The variable importance in the projection scores of volatile compounds.

A total of 56 volatile compounds were identified in the high-temperature roasted chicken marinated with water and six types of tea. The PLS-DA (Fig. 3A) analysis revealed that the high-temperature roasted chicken marinated with different kinds of tea occupied distinct quadrants and did not overlap with the control sample. Although high-temperature roasted chicken marinated with oolong tea, yellow tea, green tea, white tea, and black tea were in different quadrants, there was partial overlap in their profiles, suggesting that the differences in volatile compounds among them were not significant.

High-temperature roasting generates large amounts of aldehydes, ketones, alcohols, aromatic compounds, phenols, and hydrocarbons. The results of the heatmap for the volatile compounds in high-temperature roasted chicken marinated with different types of tea are shown in Fig. 3B, as observed, the overall volatile compounds in the high-temperature roasted chicken marinated with black tea, oolong tea, and yellow tea increased. Amidst these, the high-temperature roasted chicken marinated with yellow tea exhibited the highest content of volatile compounds. Protein, carbohydrates, amino acids, and other nutrients undergo intense Maillard reactions under high-temperature aerobic conditions. Alpha-dicarbonyl compounds engage with amino acids, undergoing degradation, resulting in aldehyde formation. The roasted chicken marinated with tea showed a lower content of nonanal, which can be attributed to the antioxidative properties of the tea marinade. Yuan et al. (2023) found that antioxidant-rich spices can attenuate the Maillard reaction and diminish the generation of volatile compounds. Alcohols primarily originate from lipid oxidation and Strecker degradation reactions (Yang et al., 2017). Compared to the control samples, the roasted chicken marinated with tea exhibited an increase of the 1-Hexanol 2-ethyl, a compound with the subtly floral. Ketones typically have a higher odor threshold compared to aldehydes. It has been reported that alkenes and alkanes represent representative flavors of fruit, spices, citrus, and sweetness (Yuan et al., 2023). After marinated with tea, their concentrations in the food were significantly enhanced. Spices are rich in flavor compounds that can transfer to chicken. During roasting, these flavor compounds in the spices break down due to heat, resulting in the unique flavors associated with roasted chicken. Additionally, water-soluble compounds from the spices seep into the chicken, offering more substrates for the Maillard reaction while roasting. Furthermore, the compounds in spices can alter the structure of myofibrillar proteins, facilitating the release of flavors from the meat (Sun, Yu, Saleh, et al., 2024).

Based on the classification of PLS-DA (Fig. 3C), compounds exhibiting VIP > 1.0 were chosen as differential volatile compounds for distinguishing high-temperature roasted chicken marinated with different types of tea. Nonanal was present in higher amounts in control samples. Nonanal exhibits aromas of roses, citrus, and possesses a strong greasy odor (Pino & Mesa, 2010). It is an important volatile compound produced through the β-oxidation of fats. In high-temperature roasted chicken marinated with black tea, the contents of 2,3,5-Trimethyl-pyrazine and Acetaldehyde were higher. 2,3,5-Trimethyl-pyrazine is a Maillard reaction product that provides a unique barbecue flavor to heat-treated foods. In high-temperature roasted chicken marinated with yellow tea, 4-Dodecene has a higher content compared to other marinades, making it the main volatile compound.

### 3.4 Hazardous compounds in chicken marinated with different teas after high-temperature roasting

The concentration of hazardous compounds in chicken roasted at high-temperature is shown in Table 2. In the control chicken samples, the ACY content was 57.56 ng/g, the 5-HMF content was 74.45 ng/g, the HCAs content was 4.66 ng/g, and the PAHs content was 34.99 ng/g. After marinating with various tea marinades, the ACY content in high-temperature roasted chicken ranges from 16.05 to 57.50 ng/g, the 5-HMF content ranges from 22.43 to 228.53 ng/g, the HCAs content ranges from 0.35 to 4.34 ng/g, and the PAHs content range from 23.66 to 32.10 ng/g. Different tea marinades showed different inhibitory effects on the generation of ACY, 5-HMF, HCAs, and PAHs. Except for white tea, other tea marinades can significantly reduce the generation of ACY. Compared to other tea marinades, green tea marinade exhibits the most effective inhibitory effect on 5-HMF, reducing its content by more than half. Oolong tea, yellow tea, green tea, and dark tea showed a substantial inhibitory impact on the formation of HCAs, achieving an inhibitory rate of nearly 50 %. Except for black tea, other tea marinades can significantly reduce the generation of PAHs, among which white tea has the best effect.

**Table 2:** Hazardous compounds content of chicken roasted at high temperature after marinating with distilled water and different tea marinades.

Hazards	Content of hazards(ng/g)						
	control	Black tea	Oolong tea	Yellow tea	Green tea	White tea	Dark tea
ACY	57.56 ± 3.25^a^	33.55 ± 3.41^b^	16.46 ± 2.86^c^	31.33 ± 1.71^b^	16.05 ± 0.91^c^	57.50 ± 9.63^a^	17.77 ± 2.75^c^
5-HMF	74.45 ± 4.45^c^	119.15 ± 14.90^b^	228.53 ± 32.38^a^	54.49 ± 10.61^cd^	22.43 ± 1.81^d^	58.08 ± 9.71^cd^	64.70 ± 3.69^c^
DMIP	0.99 ± 0.22^a^	1.02 ± 0.16^a^	0.43 ± 0.14^b^	0.20 ± 0.06^bc^	nd	0.40 ± 0.03^b^	0.07 ± 0.02^c^
PHIP	1.99 ± 0.32^a^	2.48 ± 0.63^a^	0.46 ± 0.27^b^	0.87 ± 0.38^b^	0.04 ± 0.01^b^	2.61 ± 0.44^a^	0.13 ± 0.03^b^
IQ[4,5-b]	nd	nd	nd	nd	nd	nd	nd
IQ	0.03 ± 0.01^a^	0.03 ± 0.01^a^	0.02 ± 0.01^ab^	0.02 ± 0.00^b^	0.02 ± 0.00^b^	0.02 ± 0.00^ab^	0.01 ± 0.00^b^
MEIQ	0.04 ± 0.02^a^	0.03 ± 0.01^ab^	0.03 ± 0.01^ab^	0.02 ± 0.00^bc^	0.01 ± 0.00^c^	0.02 ± 0.00^abc^	0.01 ± 0.00^c^
IQx	0.08 ± 0.01^a^	0.05 ± 0.01^b^	0.03 ± 0.01^c^	0.02 ± 0.00^cd^	nd	0.03 ± 0.00^c^	0.01 ± 0.00^de^
8-MEIQx	0.09 ± 0.03^b^	0.17 ± 0.04^a^	0.05 ± 0.04^bc^	0.03 ± 0.01^d^	nd	0.05 ± 0.01^bc^	nd
7,8-DIMEIQx	0.09 ± 0.01^a^	0.07 ± 0.03^a^	nd	0.02 ± 0.00^b^	nd	0.09 ± 0.01^a^	nd
Phe-P	nd	nd	nd	nd	nd	nd	nd
AaC	nd	nd	nd	nd	nd	nd	nd
MeAaC	0.02 ± 0.00^a^	0.02 ± 0.00^a^	0.02 ± 0.00^a^	0.02 ± 0.00^a^	nd	nd	nd
Norharman	1.01 ± 0.18^b^	1.42 ± 0.21^a^	1.47 ± 0.09^a^	0.56 ± 0.12^cd^	0.27 ± 0.03^d^	0.86 ± 0.09^bc^	0.32 ± 0.02^d^
Harman	0.31 ± 0.08^b^	0.48 ± 0.08^a^	0.49 ± 0.08^a^	0.16 ± 0.01^cd^	0.02 ± 0.00^e^	0.26 ± 0.01^bc^	0.06 ± 0.00^de^
Total HCAs	4.66 ± 1.07^a^	5.77 ± 1.41^a^	2.98 ± 0.71^bc^	1.92 ± 0.68^cd^	0.35 ± 0.06^e^	4.34 ± 0.544^ab^	0.62 ± 0.06^e^
Na	3.70 ± 0.93^a^	2.76 ± 0.34^b^	1.67 ± 0.10^c^	1.69 ± 0.55^c^	1.78 ± 0.39^c^	1.17 ± 0.10^c^	1.81 ± 0.28^c^
F	27.42 ± 1.60^a^	25.59 ± 2.08^a^	20.94 ± 1.45^bc^	24.11 ± 1.50^ab^	24.64 ± 2.68^a^	18.59 ± 1.05^c^	20.87 ± 1.31^bc^
Ant	1.85 ± 0.07^a^	1.74 ± 0.12^ab^	1.59 ± 0.01^ab^	1.58 ± 0.02^b^	1.62 ± 0.09^ab^	1.75 ± 0.18^ab^	1.73 ± 0.22^ab^
Flu	1.04 ± 0.15^ab^	0.76 ± 0.16^b^	0.86 ± 0.22^b^	0.69 ± 0.15^b^	0.83 ± 0.10^b^	1.32 ± 0.09^a^	0.99 ± 0.33^ab^
BaA	0.46 ± 0.03^b^	0.60 ± 0.03^a^	0.46 ± 0.03^b^	0.54 ± 0.10^ab^	0.53 ± 0.05^ab^	0.46 ± 0.05^b^	0.31 ± 0.04^c^
Chr	0.13 ± 0.02^bc^	0.31 ± 0.07^a^	0.23 ± 0.04^ab^	0.23 ± 0.10^ab^	0.11 ± 0.05^c^	0.10 ± 0.00^c^	0.11 ± 0.04^c^
Hazards	Content of hazards(ng/g)						
	control	Black tea	Oolong tea	Yellow tea	Green tea	White tea	Dark tea
BaA	0.46 ± 0.03^b^	0.60 ± 0.03^a^	0.46 ± 0.03^b^	0.54 ± 0.10^ab^	0.53 ± 0.05^ab^	0.46 ± 0.05^b^	0.31 ± 0.04^c^
Chr	0.13 ± 0.02^bc^	0.31 ± 0.07^a^	0.23 ± 0.04^ab^	0.23 ± 0.10^ab^	0.11 ± 0.05^c^	0.10 ± 0.00^c^	0.11 ± 0.04^c^
B[*b*]F	nd	nd	nd	nd	nd	nd	nd
B[*k*]F	0.15 ± 0.01^a^	0.14 ± 0.02^ab^	0.11 ± 0.03^abc^	0.10 ± 0.02^abc^	0.09 ± 0.02^bc^	0.07 ± 0.03^c^	0.09 ± 0.04^bc^
BaP	0.23 ± 0.02^a^	0.20 ± 0.02^ab^	0.16 ± 0.05^b^	0.18 ± 0.00^ab^	0.19 ± 0.01^ab^	0.19 ± 0.01^ab^	0.20 ± 0.02^ab^
B[*ghi*]p	nd	nd	nd	nd	nd	nd	nd
Total PAHs	34.99 ± 2.46^a^	32.10 ± 2.50^ab^	26.01 ± 1.58^cd^	29.12 ± 1.86^bc^	29.77 ± 2.31^bc^	23.66 ± 1.01^d^	26.11 ± 1.19^cd^

Results are presented as the mean ± standard deviation, *n* = 3. Values of different groups with lower-case letters (a-d) is significantly different at *P* < 0.05.

As observed from Table 2, compared with the control samples, the content of most hazardous compounds in high-temperature roasted chicken marinated with tea exhibited a decreasing trend. Among them, green tea demonstrated the best effect in inhibiting hazardous compounds. The majority of hazardous compounds are compounds with cyclic structures, and the antioxidant components in tea may interfere with the formation of these substances by reducing or quenching active free radicals (Lu et al., 2018). Applying marinades containing antioxidants from tea to processed meat products at high-temperature is an effective method to reduce the generation of hazardous compounds. Quelhas et al. (2010) found that marinating meatballs with green tea extract during the marination process can reduce the levels of PHIP and AaC. Wang et al. (2018) discovered a pronounced inhibitory effect of green tea on PAHs in marinaded chicken meat. The antioxidant compounds present in tea marinades served as radical scavengers or free radical quenchers. Additionally, the tea marinade acted as a barrier, mitigating the direct contact with the heat source, leading to the effective mitigation of hazardous compounds in high-temperature roasted chicken.

Although the marinade of tea inhibits the generation of hazardous compounds, not all types of hazardous compounds are affected, and some marinades might facilitate the formation of specific hazardous compounds. For example, 8-MEIQx in chicken roasted with black tea marinades significantly increases. Red tea, oolong tea, and yellow tea also had negative impacts on Chr. During the process of eliminating free radicals, antioxidant compounds, by providing hydrogen, can be oxidized into free radicals themselves, which may contribute to the generation of hazardous compounds (Sharma & Hajaligol, 2003). Jongberg et al. (2013) found the concentration of free radicals in steamed sausages supplemented with green tea extract is markedly higher, and the content of free radical compounds has increased accordingly. In the glucose-acrylamide model under high-temperature conditions, the addition of chlorogenic acid leads to an increase of acrylamide in the model (Cai et al., 2014). It has not been specifically explained how antioxidant compounds promote the generation of free radicals, leading to an increase in hazardous compounds. When food undergoes thermal processing, elevated temperatures speed up oxidation reactions, resulting in the creation of hazardous compounds. The antioxidant properties found in teas can help reduce the formation of hazardous compounds by slowing down these oxidation reactions. Furthermore, the hydroxyl and aromatic rings present in phenolic compounds in tea can interact with the precursors of hazardous compounds, thereby inhibiting its production (Bortolini et al., 2021; Quelhas et al., 2010).

### 3.5 Key compounds of tea marinades

**Fig. 4:**
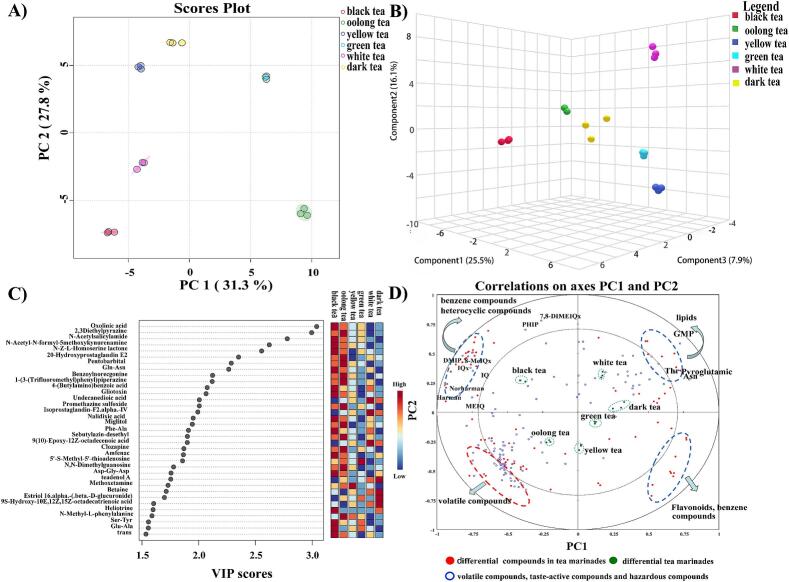
(A) PCA of compounds in different tea marinades. (B) PLS-DA of compounds in different tea marinades. (C) The variable importance in the projection scores of compounds in different tea marinades. (D) PLS regression results for differential compounds in different tea marinades and the volatile compounds, flavor compounds, and hazardous compounds in chicken roasted at high temperatures after marinating with different teas.

A comprehensive analysis revealed the detection of 252 distinct compounds in tea marinades, 36 flavonoids, 34 benzene compounds, 30 lipids, 27 amino acids and their derivatives, 19 organic acids and their derivatives, 17 oxides, 15 polyketones, 14 terpenes, 9 organic nitrogen compounds, 8 nucleosides and nucleotides, 1 alkaloid, and 2 other substances. From the score plots of PCA (Fig. 4A) and the 3D scatter plots of PLS-DA (Fig. 4B), it is evident that the various tea marinades do not overlap, demonstrating distinct differences. Different types of tea marinades are effectively discriminated against, indicating pronounced compositional disparities in the resulting tea marinades.

Based on the screening of compounds with a VIP in the project (Fig. 4C), VIP > 1.5, a total of 35 compounds causing differences were identified. These compounds include 11 heterocyclic compounds, 7 lipids, 5 organic acids and their derivatives, 3 amino acids and their derivatives, 5 benzene compounds, 2 nucleosides and nucleotides, 1 alkaloid, and 1 organic oxide. Lipid degradation is one crucial pathway in the tea production process for the generation of aroma. Carbonyl compounds resulting from lipid oxidation can engage in Maillard reactions with amino acid degradation products, giving rise to the production of flavor compounds. In comparison to other tea marinades, green tea marinades contain lower levels of important lipid compounds. This may be attributed to the processing techniques of tea leaves. During green tea processing, chlorophyll decomposes, phospholipids decrease, glycolipids degrade significantly, and the lipid content partially decreases (Li et al., 2021). Amino acids play a crucial role in imparting freshness and sweetness to the tea marinade, while organic acids significantly contribute to its sour taste. Compared with other tea marinades, black tea contains more amino acids, organic acids and their derivatives. This is attributed to the extensive fermentation process of black tea, where polyphenolic compounds undergo predominant oxidation and polymerization reactions. This may explain the variation in taste experienced when high-temperature roasted chicken is marinated with tea marinades.

PLS-R regression results for differential compounds in different tea marinades and the volatile compounds, flavor compounds, and hazardous compounds in chicken roasted at high temperatures after marinating with different teas (Fig. 4D). 77 differential compounds with VIP > 1.0 were identified among six tea marinades (x variables), 51 taste-active compounds, 56 volatile compounds, and 25 hazardous compounds (y variables) were investigated in high-temperature roasted chicken marinated with different tea marinades. The different marinades were closely grouped, indicating strong similarity among differential compounds of tea marinades and taste-active compounds, volatile compounds, and hazardous compounds in high-temperature roasted chicken. Variables nearby within the same quadrant also show higher correlations, whereas variables in the diagonal quadrant tend to exhibit negative correlations (Sherman et al., 2020). The majority of differential compounds that exhibit significant negative correlations with the y variable of volatile compounds are lipid compounds. Lipids in tea are important precursors for tea aroma constituents. The changes in lipid content and composition resulting from lipid degradation often influence the types of aroma present (Pino & Mesa, 2010). Aldehydes and alcohols derived from lipid degradation are important contributors to the fresh and floral aroma of tea. The y-variables of associated hazardous compounds exhibit significant negative correlations with flavonoid and antioxidative benzene compounds located in the fourth quadrant.

Flavonoids are a class of natural compounds found in plants with potent abilities to scavenge free radicals and inhibit oxidation reactions. In yellow tea and green tea marinades, there was a higher content of flavonoid compounds that negatively correlated with hazardous compounds. Green tea and black tea marinades had lower levels of lipid compounds that negatively correlate with volatile compounds. Yellow tea, green tea, and black tea are considered excellent choices as tea marinades.

### 3.6 Analysis of the interactive relationship between different tea marinades and high-temperature roasted chicken flavor and hazardous compounds

**Fig. 5:**
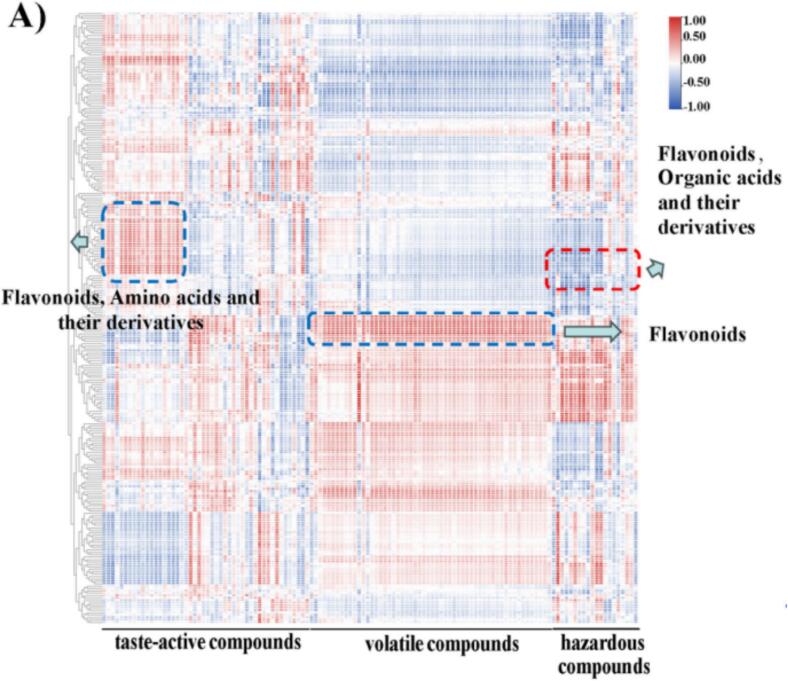
The correlation heatmap of the compounds in tea marinades and the flavor compounds, volatile compounds, and hazardous compounds in chicken roasted at high temperature after marinated with different tea.

In Fig. 5, we performed a correlation analysis between 252 compounds in tea marinades and 51 taste-active compounds, 56 volatile compounds, and 25 hazardous compounds in high-temperature roasted chicken. The flavor compounds in roasted chicken exhibited correlations with many compounds found in tea marinades, and significant correlations with amino acids, their derivatives, and flavonoid compounds were observed. This may be attributed to the interaction between flavonoid compounds and sweet taste receptors, enhancing the perception of sweetness in food and thereby influencing the flavor compounds in high-temperature roasted chicken. FAA not only provides umami and sweet taste to meat products but also acts as a flavor precursor, participating in complex biochemical reactions during meat processing. Volatile compounds in roasted chicken showed correlations with flavonoid compounds in tea marinades. The antioxidant capacity of flavonoid compounds in tea marinades delayed reactions such as lipid oxidation, thereby improving the flavor characteristics of high-temperature roasted chicken.

**Fig. 6:**
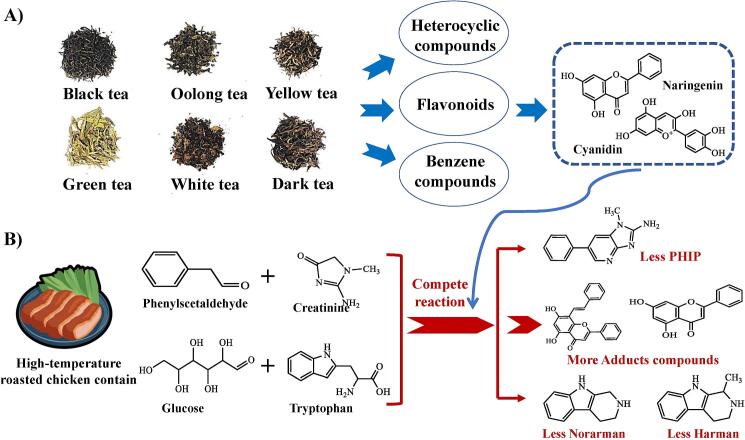
(A) Compounds inhibit the formation of hazardous compounds in tea marinades. (B) Mechanism of flavonoids inhibiting the formation of hazardous compounds in tea marinades.

Hazardous compounds in roasted chicken exhibited a negative correlation with flavonoid compounds and organic acids in tea marinades. Cheng et al. (2008) discovered that flavonoid compounds in a simulated system were found to indirectly impede the generation of PhIP by counteracting benzaldehyde. The aringenin can react with the precursor substance benzaldehyde of PHIP, forming adduct compounds, thereby inhibiting the generation of PHIP. Zhang et al. (2023) discovered that cyanidin can inhibit the generation of THCA and MTCA, thereby reducing the generation of Norharman and Harman. The reduction in the levels of hazardous compounds during the high-temperature roasting of chicken may be attributed to the presence of flavonoids such as naringin and cyanidin (Fig. 6A) in the tea marinades. These flavonoids exhibit potent capabilities in scavenging free radicals, thereby markedly restraining the generation of free radicals during the high-temperature processing of food. It contributes to the mitigation of hazardous compound formation during the high-temperature roasting of chicken and helps preserve the quality of the food product (Fig. 6B). Flavonoid compounds in tea marinades are pivotal in augmenting flavor and reducing the hazardous compounds.

## 4 Conclusion

The influence of tea marinades on both flavor and the presence of hazardous compounds in high-temperature roasted chicken were explored. Tea marinades reduced the accumulation of bitter taste-active compounds in the roasted chicken, while white tea, black tea, and green tea promoted the accumulation of distinctive umami taste-active compounds. Oolong tea and yellow tea marinades increased the total volatile compound content in high-temperature roasted chicken. Marinating the chicken with tea marinades effectively suppresses the formation of most hazardous compounds during high-temperature roasted. 252 compounds in tea marinades were explored, and differences were observed among the compounds in different tea marinades. Flavonoid compounds in tea marinades were significantly correlated with the flavor and hazardous compounds in high-temperature roasted chicken. The application of tea marinade in roasted meat has a broad prospect and potential. Tea, as a natural product found worldwide, is readily accepted by consumers. Through rational selection of tea leaves, control of marinating time and temperature, and combination with other seasonings, the flavor of roasted meat can be further enhanced and the generation of harmful substances can be inhibited, providing consumers with a more delicious and healthy roasted meat experience.

## Abbreviations

**Table:** 

ACY	acrylamide
5-HMF	5-hydroxymethylfurfural
HCAs	heterocyclic amines
PAHs	polycyclic aromatic hydrocarbons
VIP	variable importance in projection
PLS-DA	partial least square discriminant analysis
PCA	principal component analysis
PLS-R	partial least square discriminant analysis.

## CRediT authorship contribution statement

**Ji Wang:** Writing – original draft, Software, Methodology, Investigation, Formal analysis, Data curation, Conceptualization. **Jing Che:** Writing – original draft, Methodology, Data curation. **Xu-Song Wang:** Methodology, Investigation. **Lei Qin:** Writing – review & editing, Project administration, Funding acquisition. **Xu-Hui Huang:** Writing – review & editing, Validation, Supervision, Software, Resources, Methodology, Investigation.

## Declaration of competing interest

The authors declare that they have no known competing financial interests or personal relationships that could have appeared to influence the work reported in this paper.

## Data Availability

Data will be made available on request.
